# Renal Fungal Balls - The Importance of Radiological Findings

**DOI:** 10.1590/0037-8682-0558-2019

**Published:** 2020-03-16

**Authors:** Vanessa Barcelos, Ana Carolina Ferreira

**Affiliations:** 1 Hospital do Divino Espírito Santo de Ponta Delgada, Ponta Delgada, Portugal.

**FIGURE 1:**
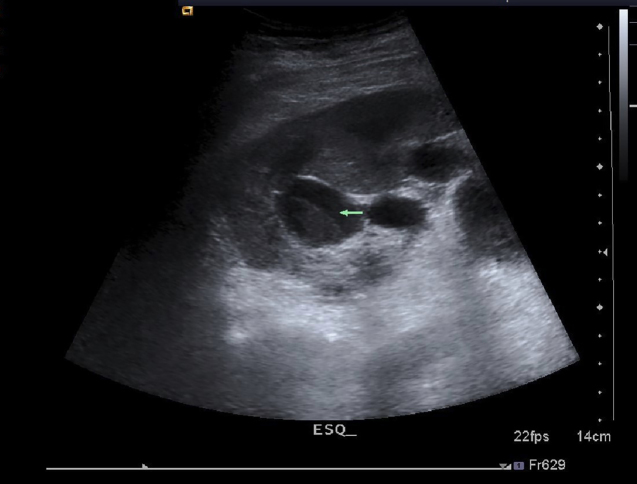
Ultrasonogram of the left kidney showing a hyperechoic mass in the renal calyx with no posterior shadow cone.

A 74-year-old diabetic woman presented to the Emergency Department with a high fever and prostration. She had been recently admitted three times for acute urinary retention resulting from an infection with negative urine culture. Laboratory tests at admission showed high C-reactive protein level (16.7 mg/dL), impaired renal function (Cr 2.42 mg/dL), and pus cells (>70/ high-power field). A kidney ultrasound (Figure 1) revealed heterogeneous deposits in both upper renal calyces with no color flow in the Doppler study. After consulting the radiologist, we suspected *fungus balls*. 

The blood cultures were negative and the urine culture yielded fluconazole-resistant *Candida glabrata*. The patient was administered intravenous caspofungin and referred for bilateral nephrostomies with amphotericin instillations. Systemic amphotericin B was to be avoided to prevent further deterioration of her renal function. While under medical treatment, the patient refused surgical treatment and died. 

Upper urinary tract fungal infections are uncommon and fungal bezoar formation is particularly rare^1^.

Awareness of the various radiological findings of this rare clinical entity and an increased suspicion in high-risk individuals will help to overcome the challenges related to early diagnosis and proper treatment^2^.
